# Spectrum of cutaneous adverse reactions to aromatic antiepileptic drugs and *human leukocyte antigen* genotypes in Thai patients and meta-analysis

**DOI:** 10.1038/s41397-021-00247-3

**Published:** 2021-06-26

**Authors:** Chonlaphat Sukasem, Suthida Sririttha, Chonlawat Chaichan, Thapanat Nakkrut, Patompong Satapornpong, Kanoot Jaruthamsophon, Thawinee Jantararoungtong, Napatrupron Koomdee, Sadeep Medhasi, Sarawut Oo-Puthinan, Ticha Rerkpattanapipat, Jettanong Klaewsongkram, Pawinee Rerknimitr, Papapit Tuchinda, Leena Chularojanamontri, Napatra Tovanabutra, Naravut Suvannang, Thanyada Rungrotmongkol, Surasak Saokaew, Wichai Aekplakorn, Apichaya Puangpetch

**Affiliations:** 1 Division of Pharmacogenomics and Personalized Medicine, Department of Pathology, Faculty of Medicine Ramathibodi Hospital, Mahidol University, Bangkok, Thailand; 2 Laboratory for Pharmacogenomics, Somdech Phra Debaratana Medical Center (SDMC), Ramathibodi Hospital, Bangkok, Thailand; 3 The Thai Severe Cutaneous Adverse Drug Reaction (THAI-SCAR) research group, Bangkok, Thailand; 4 Pharmacogenomics and Precision Medicine, The Preventive Genomics & Family Check-up Services Center, Bumrungrad International Hospital, Bangkok, Thailand; 5 Department of Clinical Pharmacy Practice, Faculty of Pharmacy, Mahidol University, Bangkok, Thailand; 6 Department of Pathology, School of medicine, University of Phayao, Phayao, Thailand; 7 Department of Pharmacy Practice, Faculty of Pharmaceutical Sciences, Naresuan University, Phitsanulok, Thailand; 8 Department of Pharmacy, Neurological Institute of Thailand, Bangkok, Thailand; 9 Division of General Pharmacy Practice, Department of Pharmaceutical Care, College of Pharmacy, Rangsit University, Pathum Thani, Thailand; 10 Department of Pathology, Faculty of Medicine, Prince of Songkla University, Songkla, Thailand; 11 Department of Microbiology and Immunology, Faculty of Tropical Medicine, Mahidol University, Bangkok, Thailand; 12 Division of Allergy Immunology and Rheumatology, Department of Medicine, Faculty of Medicine, Ramathibodi Hospital, Mahidol University, Bangkok, Thailand; 13 Skin and Allergy Research Unit, Division of Allergy and Clinical Immunology, Department of Medicine, Faculty of Medicine, Chulalongkorn University, Bangkok, Thailand; 14 Division of Dermatology, Department of Medicine, Faculty of Medicine, Skin and Allergy Research Unit, Chulalongkorn University, Bangkok, Thailand; 15 Department of Dermatology, Faculty of Medicine Siriraj Hospital, Mahidol University, Bangkok, Thailand; 16 Division of Dermatology, Department of Internal Medicine, Chiang Mai University, Chiang Mai, Thailand; 17 Pre-Clinical Laboratory, Triamwitpattana school, Bangkok, Thailand; 18 Structural and Computational Biology Research Unit, Department of Biochemistry, Faculty of Science and Program in Bioinformatics and Computational Biology, Faculty of Science, Chulalongkorn University, Bangkok, Thailand; 19 Division of Pharmacy Practice, Department of Pharmaceutical Care, School of Pharmaceutical Sciences, University of Phayao, Phayao, Thailand; 20 Center of Health Outcomes Research and Therapeutic Safety (Cohorts), School of Pharmaceutical Sciences, University of Phayao, Phayao, Thailand; 21 Unit of Excellence on Clinical Outcomes Research and IntegratioN (UNICORN), School of Pharmaceutical Sciences, University of Phayao, Phayao, Thailand; 22 Department of Community Medicine, Ramathibodi Hospital, Mahidol University, Bangkok, Thailand

**Keywords:** Prognosis, Prognostic markers, Prognostic markers

## Abstract

Aromatic antiepileptic drugs (AEDs)-induced cutaneous adverse drug reactions (cADRs) add up to the limited use of the AEDs in the treatment and prevention of seizures. *Human leukocyte antigen-B* (*HLA-B*) alleles have been linked to AEDs-induced cADRs. We investigated the association between cADRs (including Stevens–Johnson syndrome; SJS/toxic epidermal necrolysis; TEN, drug reaction with eosinophilia and systemic symptoms; DRESS, and Maculopapular eruption; MPE) caused by AEDs (phenytoin, carbamazepine, lamotrigine, phenobarbital and oxcarbazepine) and *HLA-B* alleles in Thai population. Through the case-control study, 166 patients with AEDs-induced cADRs, 426 AEDs-tolerant patients (AEDs-tolerant controls), and 470 healthy subjects (Thai population) were collected. The *HLA* genotypes were detected using the polymerase chain reaction-sequence specific oligonucleotide probe (PCR-SSOP) method. We also performed a meta-analysis with these data and other populations. The carrier rate of HLA-B*15:02 was significantly different between AEDs-induced cADRs group and AEDs-tolerant group (Odds ratio; OR 4.28, 95% Confidence interval; CI 2.64–6.95, *p* < 0.001), AEDs-induced cADRs group and Thai population (OR 2.15, 95%CI 1.41–3.29, *p* < 0.001). In meta-analysis showed the strong association HLA-B*15:02 with AEDs-induced cADRs (OR 4.77, 95%CI 1.79–12.73, *p* < 0.001). Furthermore, HLA-B*15:02 was associated with SJS/TEN induced by AEDs (OR 10.28, 95%CI 6.50–16.28, *p* < 0.001) Phenytoin (OR 4.12, 95%CI 1.77–9.59, *p* = 0.001) and carbamazepine (OR 137.69, 95%CI 50.97–371.98, *p* < 0.001). This study demonstrated that genetic association for AEDs-induced cADRs was phenotype-specific. A strong association between HLA-B*15:02 and AEDs-induced SJS/TEN was demonstrated with an OR of 10.79 (95%CI 5.50–21.16, *p* < 0.001) when compared with AEDs-tolerant group. On the other hand, the carrier rates of HLA-B*08:01, HLA-B*13:01, and HLA-B*56:02 were significantly higher in the DRESS group compared with the AEDs-tolerant group (*p* = 0.029, 0.007, and 0.017, respectively). The HLA-B*15:02 allele may represent a risk factor for AEDs-induced cADRs.

## Introduction

Aromatic antiepileptic drugs (AEDs), such as phenytoin (PHT), carbamazepine (CBZ), lamotrigine (LTG), phenobarbital (PB), and oxcarbazepine (OXC), commonly prescribed to prevent or treat seizure, are frequently associated with cutaneous adverse drug reactions (cADRs) [1]. The overall incidence of cADRs induced by AEDs is ~10% [2]. cADRs can be classified into two types, immediate and delayed type. cADRs associated with AEDs are usually delayed type, and clinical manifestations include from mild maculopapular eruption (MPE) to severe and life-threatening severe cutaneous adverse reactions (SCARs); Stevens–Johnson syndrome (SJS), toxic epidermal necrolysis (TEN), and hypersensitivity syndrome (HSS)/drug-induced hypersensitivity syndrome (DIHS)/drug reaction with eosinophilia and systemic symptoms (DRESS) [3].

MPE, the mild delayed type cADRs, presents as a widespread, symmetrically distributed rash composed of pink-to-red macules and papules that may coalesce to form plaques. Redness of skin without blister, pruritus and low-grade fever may occur in MPE, but does not involve the internal organs [4]. SJS and TEN are severe life-threatening delayed type cADRs characterized by febrile illness in acute phases followed by the predominant involvement of skin and mucous membrane necrosis and systemic symptoms. The degree of skin detachment expressed in terms of the % body surface area affected classifies the SJS and TEN into 3 types: <10% body surface area is affected in SJS, 10–30% body surface area is affected in SJS-TEN overlap, and >30% body surface area is affected in TEN [5]. DRESS has a wide range of clinical characteristics, such as cutaneous eruption, fever, lymphadenopathy, hepatitis, and hematologic abnormalities with eosinophilia and atypical lymphocytes [6]. Severe cADRs has a crucial burden on healthcare system with the mortality rates reaching up to 40% [7]. Alleles of the human leukocyte antigen (*HLA*) genes have been associated with cADRs, and the screening of *HLA* alleles prior to the administration of AEDs can prevent the occurrence of cADRs.

The *HLA-B* genotype is a crucial immunological risk factor for cADRs. In 2004, Chung et al. reported a strong association between the HLA-B*15:02 allele and CBZ-induced SJS/TEN in Han Chinese [8]. In 2013, a systematic review and meta-analysis in Thai and Malaysian populations validated the association between the HLA-B*15:02 allele and CBZ-induced SJS/TEN [9]. In Thai population, the HLA-B*15:02 allele has a frequency of 8.16% which is higher when compared to other ethnicities: 4.87% in Asians, 2% in African-American, and rarely observed in East Asian populations (Japanese and Korean), Caucasian, and Hispanic/North Americans [10, 11]. The aim of this study was to extend the investigation of *HLA-B* susceptibility to AEDs-induced cADRs in Thai patients.

## Materials and methods

### Subjects and characteristics

We recruited cases between 2011 and 2017 with AEDs-induced cADRs. The enrolled cases of 166 patients with AEDs-induced cADRs included 71 with MPE, 49 with DRESS, and 46 with SJS/TEN. Patients tolerated to various AEDs (AEDs-tolerant controls; *n* = 426) for at least 6 months without evidence of cutaneous adverse effects were also recruited in this study. Healthy subjects (Thai population; *n* = 470) who did not take AEDs were included for analysis. All individuals enrolled were unrelated Thai population. This study was approved by the Ramathibodi Hospital Ethical Review Board for Human Research. Written informed consent was obtained from all the participants.

### Diagnosis of AEDs-induced cADRs

All patients were diagnosed and confirmed based on the photographs, pathological slides, clinical morphology, and medical records by a dermatologist and allergist. MPE was diagnosed as a macular and papular rash without skin detachment or organ involvement. The RegiSCAR criteria were used to diagnose and classify SCARs [12]. DRESS was characterized by acute skin rash, fever above 38 °C, facial edema, enlarged lymph nodes, internal organ involvement (e.g., liver, kidney and lung), and hematological abnormalities, including lymphocytosis or lymphocytopenia, eosinophilia, and thrombocytopenia [12]. SJS was defined as skin detachment of <10% of the body surface area. TEN was defined as skin detachment >30%. The SJS-TEN overlap was defined as skin detachment of 10% to 30% [3]. In some cases, in vitro assay (such as ELISpot) was performed.

### DNA extraction, *HLA-A*, *HLA-B* and CYP2C9*3 genotyping

Blood samples were collected into EDTA tubes. Genomic DNA was extracted by the MagNA Pure automated compact machine (Roche Diagnostics, USA). The purity and concentration of genomic DNA were detected using the Nanodrop ND-1000 ultraviolet spectrophotometer. DNA was stored at −20 °C before analysis.

*HLA-A* and *HLA-B* genotyping was performed using the polymerase chain reaction-sequence specific oligonucleotide probe (PCR-SSOP) method combined with Luminex™ Multiplex technology. Data analysis for the *HLA-A* and *HLA-B* assays was performed with software package HLA fusion 2.0.

TaqMan® SNP Genotyping Assays of CYP2C9*3 (1075A > C, rs1057910) were performed on the ViiA™ 7 real time-PCR System (Applied Biosystems, Foster City, California), according to the manufacturer’s instructions.

### Statistical analysis

Statistical analyses were performed using SPSS version 16.0. Means and standard deviations (SD) were calculated for all continuous data. Comparison of continuous variables among clinical characteristics between cases and controls were analyzed using the *t*-test. Chi-square test or Fisher’s exact test was used for the comparison of *HLA-A, HLA-B* and *CYP2C9* allele frequency in each group. Odds ratio (OR) and 95% confidence interval (CI) values were obtained. Bonferroni correction was applied to adjust for multiple comparisons (*P* < 0.0025) to indicate statistically significant association. For the meta-analysis, we used *“metan”* command of STATA program for pooling effect-size (StataCorp LLC. College Station, TX).

## Results

### Cases and controls study

Pharmacogenetic study was performed in 166 patients who had AEDs-induced cADRs, 426 AEDs-tolerant controls and 470 Thai population. Table 1 shows different types of cADRs and their causative AEDs including PHT (103; 62.04%), CBZ (38; 22.89%), LTG (15; 9.03%), PB (7; 4.21%) and OXC (3; 1.30%). Overall, 166 cases of AEDs-induced cADRs were 71 MPE, 46 SJS/TEN and 49 DRESS cases. Among 71 AEDs-induced MPE, we found 41(57.74%), 16(22.53%), 11(15.49%) and 3(4.22%) cases received PHT, CBZ, LTG and PB, respectively. In a group of SJS/TEN, 46 cases were related with PHT (22; 47.82%), CBZ (17; 36.95%), LTG (3; 6.52%), PB (1; 2.17%) and OXC (3; 6.52%). In a group of DRESS, the most common culprit drugs were PHT (40; 81.63%).

**Table 1:** Number of subjects in each drug group divided by phenotype of cADRs.

Drugs	MPE (*n* = 71)	SJS/TEN (*n* = 46)	DRESS (*n* = 49)	Total AEDs cases (*n* = 166)	AEDs tolerant controls (*n* = 426)
PHT	41 (57.74%)	22 (47.82%)	40 (81.63%)	103 (62.04%)	105 (24.65%)
CBZ	16 (22.53%)	17 (36.95%)	5 (10.2%)	38 (22.89%)	271 (63.61%)
LTG	11 (15.49%)	3 (6.52%)	1 (2.04%)	15 (9.03%)	50 (11.73%)
PB	3 (4.22%)	1 (2.17%)	3 (6.12%)	7 (4.21%)	–
OXC	–	3 (6.52%)	–	3 (1.30%)	–

*PHT* phenytoin, *CBZ* carbamazepine, *LTG* lamotrigine, *PB* phenobarbital, *OXC* oxcarbazepine, *AEDs* aromatic antiepileptic drugs, *SJS/TEN* Stevens–Johnson syndrome/toxic epidermal necrolysis, *DRESS* drug reaction with eosinophilia and systemic symptoms, *MPE* maculopapular eruption.

Of the AEDs tolerant controls, 105 (24.65%) patients received PHT, 271 (63.61%) patients received CBZ, and 50 (11.73%) patients received LTG. None of the AEDs tolerant controls received PB or OXC.

### Association of *HLA-B* alleles with AEDs-induced cADRs

The association between *HLA-B* alleles and AEDs-induced cADRs was evaluated by comparing the AEDs-induced cADRs group (*n* = 166) with the AEDs-tolerant controls group (*n* = 426) and the Thai population (*n* = 470). The *HLA-B* genotypes of cADRs cases induced by AEDs are shown in Table 2. We found that 46 patients of 166 cADRs cases (27.7%) carried the HLA-B*15:02 allele, while only 35 (8.21%) of AEDs-tolerant group and 71 (15.1%) of the 470 Thai population carried this allele. HLA-B*15:02 allele was statistically significant in the AEDs-induced cADRs, with respect to the AEDs tolerant group (OR 4.282, 95%CI: 2.637–6.954; *p* < 0.001) and the Thai population (OR 2.154, 95%CI: 1.411–3.290; *p* < 0.001). Furthermore, 21 patients (12.6%) AEDs-induced cADRs group were positive for HLA-B*51:01, while only 29 (6.8%) of AEDs-tolerant controls group carried this allele (OR 2.292, 95%CI: 1.311–4.007; *p* = 0.003). However, the significant associations the HLA-B*51:01 alleles disappeared after correction for multiple testing (*P* < 0.0025). Remarkably, the HLA-B*13:01 allele was present in 4 (100%) patients with PB-induced SCARs.

**Table 2:** The genotype frequency of *HLA-B* of all AEDs-induced cADRs.

*HLA-B* alleles	All AEDs induced cADRs (*n* = 166)	All AEDs tolerant control (*n* = 426)	Thai population (*n* = 470)	All AEDs-induced cADRs cases *vs* tolerant controls		All AEDs-induced cADRs cases *vs* Thai populations	
				OR (95% CI)	*p*-value	OR (95% CI)	*p*-value
B*08:01	3 (1.80%)	1 (0.23%)	3 (0.63%)	7.822 (0.808–75.74)	0.069	2.865 (0.573–14.336)*	0.186
B*13:01	28 (16.8%)	59 (13.8%)	54 (11.48%)	1.262 (0.773–2.061)	0.352	1.563 (0.952–2.565)	0.075
B*15:02	**46** (**27.7%)**	**35** (**8.21%)**	**71** (**15.1%)**	**4.282** (**2.637–6.954)**	<0.001*	**2.154** (**1.411–3.290)**	<0.001*
B*15:21	3 (1.8%)	7 (1.64%)	2 (0.42%)	1.107 (0.281–4.312)	1.000	4.242 (0.703–25.616)*	0.118
B*51:01	21 (12.6%)	29 (6.8%)	40 (8.51%)	2.292 (1.311–4.007)	0.003*	1.557 (0.889–2.728)	0.119
B*56:02	3 (1.8%)	3 (0.70%)	1 (0.21%)	2.595 (0.518–12.989)	0.357	8.632 (0.892–83.566)*	0.057

Bold values indicate statistical significance.

*AEDs* aromatic antiepileptic drugs, *cADRs* cutaneous adverse drug reactions, *HLA* human leukocyte antigen, *OR* odds ratio, *95%CI* 95% confidence interval.

*p-value less than 0.0025.

### Association of HLA-B*15:02 allele in each AEDs-drug with tolerant control

The HLA-B*15:02 allele of cADRs cases per drug is shown in Table 3. The HLA-B*15:02 frequency was statistically significant in the CBZ-induced cADRs (17 carriers of 38 cases, 44.73%) (OR 19.134, 95%CI: 7.943–46.091; *p* < 0.001) and in the LTG-induced cADRs (6 carriers of 15 cases, 40.00%) (OR 4.889, 95%CI: 1.281–18.664; *p* = 0.024) with respect to the AEDs-tolerant group. The frequency of HLA-B*15:02 allele was not significantly higher in the cases of PHT-induced cADRs compared with the tolerant controls (*p* = 0.671). One patient (14.28%) of PB- and two patients (66.67%) of OXC-induced cADRs group was a carrier of HLA-B*15:02 allele.

**Table 3:** The association of HLA-B*15:02 alleles in each drug group with tolerant control.

Drugs	cADRs (*n* = 166)	Tolerant controls (*n* = 426)	OR (95% CI)	*p*-value
PHT	20/103 (19.4%)	18/105 (17.1%)	1.165 (0.576–2.355)	0.671
CBZ	**17/38** (**44.73%)**	**11/271 (4.05%)**	**19.134 (7.943–46.091)**	<0.001*
LTG	**6/15** (**40.00%)**	**6/50 (12.00%)**	**4.889 (1.281–18.664)**	0.024*
PB	1/7 (14.28%)	–	–	–
OXC	2/3 (66.67%)	–	–	–

Bold values indicate statistical significance.

*PHT* phenytoin, *CBZ* carbamazepine, *LTG* lamotrigine, *PB* phenobarbital, *OXC* oxcarbazepine, *cADRs* cutaneous adverse drug reactions, *OR* odds ratio, *95%CI* 95% confidence interval.

*p-value less than 0.0025.

### Association between *HLA-B* alleles and AEDs-induced MPE

We compared the *HLA-B* alleles frequency of 71 AEDs-induced MPE with 426 tolerant controls, and 470 healthy controls; the results are summarized in Table 4. The carrier rate of the HLA-B*15:02 allele was higher in the MPE group than in the controls. We found that 19 of the 71 MPE cases (26.8%) carried the HLA-B*15:02 allele, while only 8.2% of the AEDs-tolerant group (OR 4.301, 95%CI: 2.294–8.064; *p* < 0.001) and 15.1% of the healthy subjects carried the allele.

**Table 4:** Association of *HLA-B* alleles with all AEDs-induced MPE.

*HLA-B* alleles	All AEDs induced MPE (*n* = 71)	All AEDs tolerant control (*n* = 426)	Thai population (*n* = 470)	All AEDs-induced MPE cases *vs* tolerant controls		All AEDs- induced MPE cases *vs* Thai populations	
				OR (95% CI)	*p*-value	OR (95% CI)	*p*-value
B*08:01	1 (1.4%)	1 (0.23%)	3 (0.63%)	5.071 (0.375–98.190)	0.266	2.224 (0.228–21.678)	0.431
B*13:01	7 (9.9%)	59 (13.8%)	54 (11.48%)	0.686 (0.300–1.569)	0.369	0.843 (0.367–1.933)	0.686
B*15:02	**19** (**26.8%)**	**35** (**8.2%)**	**71** (**15.1%)**	**4.301** (**2.294–8.064)**	**<0.001**	**2.053** (**1.146–3.678)**	**0.014**
B*15:21	1 (1.4%)	7 (1.64%)	2 (0.42%)	0.855 (0.104–7.057)	1.000	3.343 (0.299–37.352)	0. 345
B*51:01	9 (12.7%)	29 (6.8%)	40 (8.51%)	1.987 (0.898–4.398)	**0.085**	1.560 (0.722–3.372)	0. 254

Bold values indicate statistical significance.

*AEDs* Aromatic epileptic drugs, *HLA* Human leukocyte antigen, *MPE* maculopapular eruption, *OR* odds ratio, *95%CI* 95% confidence interval.

*p-value less than 0.0025.

### Association between *HLA-B* alleles and AEDs-induced SJS/TEN

We compared the *HLA-B* alleles frequency of 46 AEDs-induced SJS/TEN with 426 tolerant controls, and 470 healthy controls; the results are summarized in Table 5. The carrier rate of the HLA-B*15:02 allele was higher in the SJS/TEN group and reached a statistical significance compared to the tolerant controls (OR 10.790, 95%CI: 5.502–21.163; *p* < 0.001) and healthy subjects (OR 5.151, 95%CI: 2.740–9.684; *p* < 0.001). Moreover, HLA-B*15:21 allele showed significant association between AEDs-induced SJS/TEN and general Thai population (2/46 (4.3%) *vs* 2/470 (0.42%), OR 10.636, 95%CI: 1.462–77.359; *p* < 0.042), while the association between HLA-B*15:21 allele and AEDs-tolerant controls did not reach statistical significance (*p* = 0.214).

**Table 5:** Association of *HLA-B* alleles with all AEDs-induced SJS/TEN.

*HLA-B* alleles	All AEDs induced SJS/TEN (*n* = 46)	All AEDs tolerant control (*n* = 426)	Thai population (*n* = 470)	All AEDs-induced SJS/TEN cases *vs* tolerant controls		All AEDs- induced SJS/TEN cases *vs* Thai populations	
				OR (95% CI)	*p-*value	OR (95% CI)	*p*-value
B*13:01	7 (15.2%)	59 (13.8%)	54 (11.48%)	1.116 (0.477–2.613)	0.799	1.383 (0.589–3.245)	0.455
B*15:02	**22** (**47.8%)**	**35** (**8.2%)**	**71** (**15.1%)**	**10.790** (**5.502–21.163)**	<0.001*	**5.151** (**2.740–9.684)**	<0.001*
B*15:21	2 (4.3%)	7 (1.64%)	2 (0.42%)	2.734 (0.551–13.566)	0.214	10.636 (1.462–77.359)	0.042*
B*51:01	6 (13.0%)	29 (6.8%)	40 (8.51%)	2.053 (0.804–5.242)	0.136	1.612 (0.644–4.035)	0.282

Bold values indicate statistical significance.

*AEDs* Aromatic epileptic drugs, *HLA* Human leukocyte antigen, *SJS/TEN* Steven Johnson syndrome and toxic epidermal necrolysis, *OR* odds ratio, *95%CI* 95% confidence interval.

*p-value less than 0.05.

### Association between *HLA-B* alleles and AEDs-induced DRESS

We compared the *HLA-B* alleles frequency of 49 AEDs-induced DRESS with 426 tolerant controls, and 470 healthy controls; the results are summarized in Table 6. The carrier rates of HLA-B*08:01, HLA-B*13:01, and HLA-B*56:02 were higher in the DRESS group than in the tolerant group. The carrier rates of these alleles between the two groups reached statistical significance (*p* = 0.029, 0.007, and 0.017, respectively). The carrier rate of HLA-B*08:01 allele was not found to be significantly higher in the DRESS cases as compared to the healthy subjects (*p* = 0.052). The carrier rates for HLA-B*13:01 and HLA-B*56:02 alleles were statistically higher in the DRESS group compared to the healthy population (*p* = 0.010 and 0.002, respectively). However, the significant associations these alleles disappeared after correction for Bonferroni correction (*P* < 0.0025).

**Table 6:** Association of *HLA-B* alleles with all AEDs-induced DRESS.

*HLA-B* alleles	All AEDs induced DRESS (*n* = 49)	All AEDs tolerant control (*n* = 426)	Thai population (*n* = 470)	All AEDs-induced DRESS cases *vs* tolerant controls		All AEDs- induced DRESS cases *vs* Thai populations	
				OR (95% CI)	*p*-value	OR (95% CI)	*p*-value
B*08:01	2 (4.1%)	1 (0.23%)	3 (0.63%)	18.085 (1.609–203.238)	0.029*	8.193 (1.328–50.540)	0.052
B*13:01	**14** (**28.6%)**	**59** (**13.8%)**	**54** (**11.48%)**	**2.488** (**1.263–4.902)**	0.007*	**2.922** (**1.381–6.184)**	0.010*
B*15:02	5 (10.2%)	35 (8.2%)	71 (15.1%)	1.269 (0.473–3.408)	0.589	0.456 (0.137–1.518)	0.19
B*15:21	6 (12.2%)	29 (6.8%)	40 (8.5%)	1.910 (0.751–4.859)	0.158	1.194 (0.405–3.526)	0.767
B*56:02	**3** (**6.12%)**	**3** (**0.70%)**	**1** (**0.21%)**	**9.19** (**1.804–46.885)**	0.017*	**38.027** (**3.859–374.684)**	0.002*

Bold values indicate statistical significance.

*AEDs* Aromatic epileptic drugs, *HLA* Human leukocyte antigen, DRESS drug reaction with eosinophilia and systemic symptoms, OR odds ratio, 95%CI 95% confidence interval.

*p-value less than 0.05.

### Association between *HLA-A* and CBZ-induced DRESS and MPE

To clarify the relationship between HLA-A*31:01 and CBZ-induced DRESS and MPE, the *HLA-A* was genotyped in this study. Then, we compared the frequency of *HLA-A* alleles between the 5 CBZ-induced DRESS or 16 CBZ-induced MPE patients and the 470 normal Thai subjects. The HLA-A*31:01 allele, which was previously reported to be associated with CBZ-induced DRESS and MPE, was not found in our study. Conversely, 3 of 5 (60.00%) CBZ-induced DRESS and 6 of 16 (37.5%) CBZ-induced MPE patients were HLA-A*33:03 carriers. However, there was no significant difference of HLA-A*33:03 between patients with CBZ-induced DRESS (*p* = 0.0605) and CBZ-induced MPE (*p* = 0.1253) and normal Thai subjects (99/470; 21.06%) in our study.

### Association between CYP2C9*3 and PHT

To date there have been relatively few studies confirming the CYP2C9*3 risk association and PHT-induced SCARs. In this study we genotyped the CYP2C9*3 in 103 PHT-induced cADRs cases. The carrier rate of the CYP2C9*3 in this study was higher in the cADRs (12/103; 11.65%), which were 22.73% of SJS/TEN (5/22), 12.50% of DRESS (5/40) and 4.89% of MPE (2/41) group than in the tolerant control group (6/105; 5.71%). There was no difference in the frequency of subjects with the CYP2C9*3 between the cADRs and tolerant control group (OR 1.79; 95%CI 0.6116–5.2339, *p* = 0.2881). Nevertheless, there was also a trend toward an association between PHT-induced SJS/TEN and CYP2C9*3, compared to the tolerant control group (OR 3.88; 95%CI 0.9907–15.2146, *p* = 0.0516). Moreover, no significant differences in CYP2C9*3 frequency were found between the PHT-induced DRESS (OR 1.88; 95%CI 0.5024–7.0773, *p* = 0.3472) and PHT-induced MPE (OR 0.53; 95%CI 0.0617–4.5929, *p* = 0.5663), when compared with tolerant control group.

### Meta-analysis: HLA-B*15:02 and AEDs-induced cADRs

**Fig. 1:**
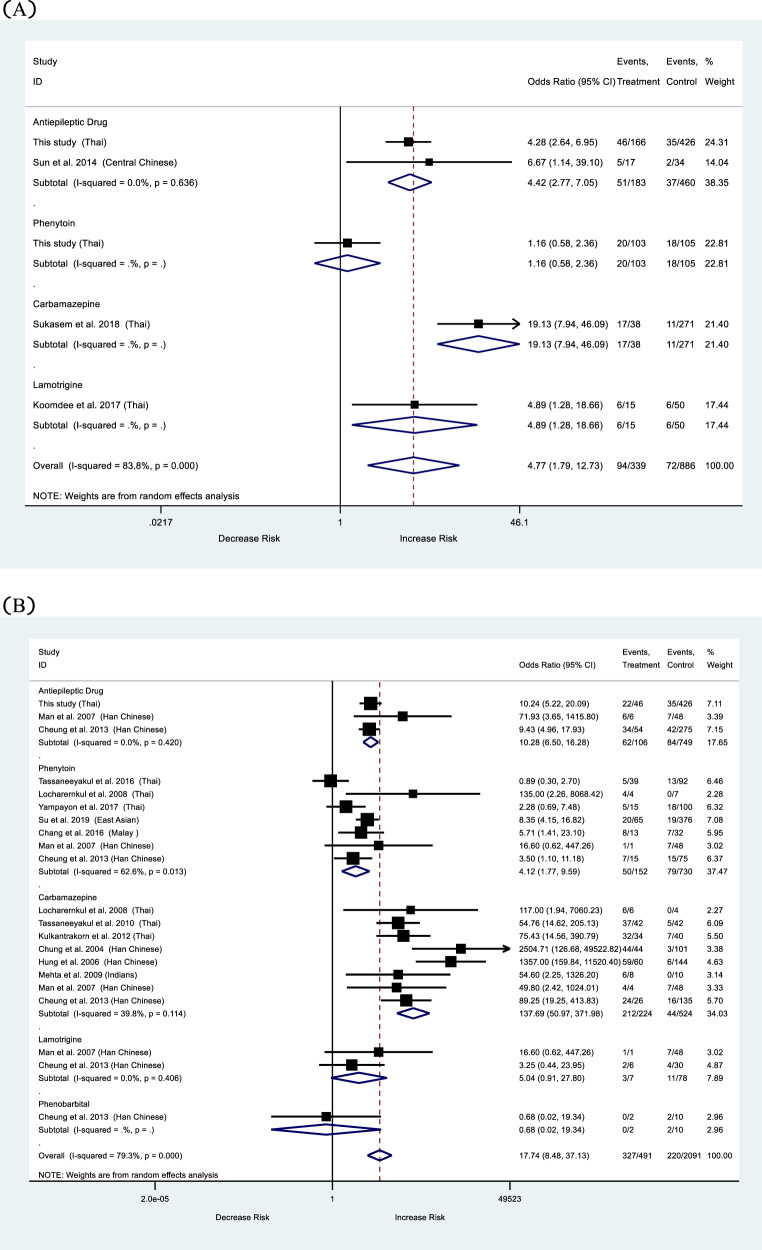

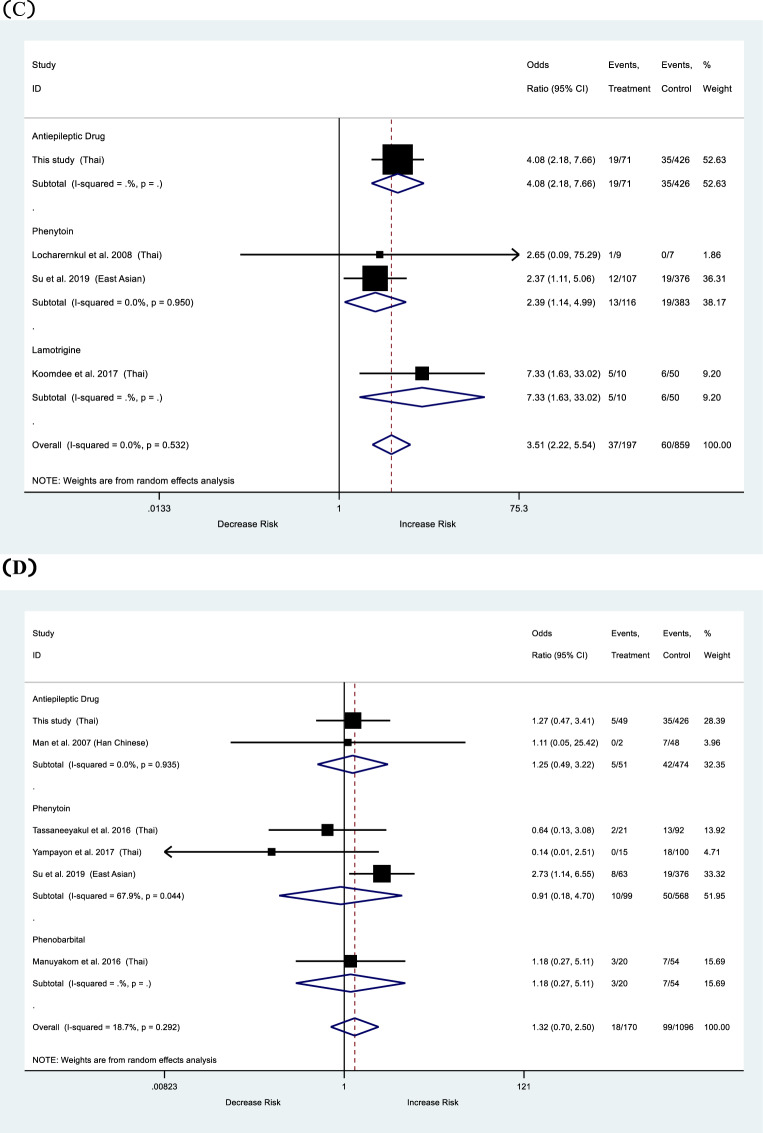
Association of HLA-B*15:02 alleles and outcomes according to antiepileptic drugs (AEDs). **A** Cutaneous adverse drug reactions (cADRs). **B** Stevens–Johnson syndrome/toxic epidermal necrolysis (SJS/TEN). **C** Maculopapular eruption (MPE). **D** Drug reaction with eosinophilia and systemic symptoms (DRESS).

The forest plots are shown in Fig. 1. Previous case-control studies on the association between HLA-B*15:02 and AEDs-induced cADRs were recruited in meta-analysis. We found that the association between the HLA-B*15:02 allele and AEDs-induced cADRs was statistically significant (OR 4.77, 95%CI 1.79–12.73, *p* < 0.001). After analyzed each AEDs separately, we found that the statistical significance of association between HLA-B*15:02 and CBZ-induced cADRs (OR 19.13, 95%CI7.94–46.09, *p* < 0.001), and HLA-B*15:02 and LTG-induced cADRs (OR 4.89, 95%CI 1.28–18.66, *p* = 0.020). But no statistical significance was found in PHT.

For SJS/TEN outcome, we found that individuals using AEDs and carrying HLA-B*15:02 allele increase of SJS/TEN by 17.74 folds compare with tolerance control group (OR 17.74, 95%CI 8.48–37.13, *p* < 0.001). We analyzed each AEDs separately and found the association of HLA-B*15:02 and PHT-induced SJS/TEN (OR 3.60, 95%CI 1.59–8.15, *p* = 0.001), and HLA-B*15:02 and CBZ-induced SJS/TEN (OR 141.89, 95%CI 48.14–419.19, *p* < 0.001).

We also found that the association between the HLA-B*15:02 allele and AEDs-induced MPE was statistically significant (OR 3.51, 95%CI 2.22–5.54, *p* < 0.001).

## Discussion

AEDs are effective for controlling seizures. Several studies have reported about the CBZ-induced SJS/TEN in Asian ancestry, including Han Chinese, Indian, Thai, Vietnamese and Malaysian [8, 13–17]. The HLA-B*15:02 allele was reported to have been associated with PHT-induced SJS/TEN, LTG-induced SJS/TEN, OXC-induced SJS/TEN. Because of the sample size limitation, association of the HLA-B*15:02 allele with these AEDs-induced cADRs is unclear and needs to be further investigated. The US-FDA has recommended the testing for HLA-B*15:02 in patients of Asian and HLA-A*31:01 in patients of European, Korean and Japanese ancestry prior to CBZ treatment due to the risk of developing SCARs [18]. Physicians avoid using all types of AEDs when the patient is HLA-B*15:02 or HLA-A*31:01 positive. Thus, we aim to investigate the association between *HLA-A, HLA-B* alleles and CYP2C9*3 genotype and AEDs-induced cADRs. We also performed a meta-analysis with these data and other populations.

In this study, we recruited cases of AEDs-induced cADRs (*n* = 166) and performed *HLA-B* allele association to find a genetic marker to screen the patients before initiating the AEDs treatment. The carrier rate of the HLA-B*15:02 and HLA-B*51:01 alleles was significantly higher in the AEDs-induced cADRs cases than the tolerant control group. HLA-B*15:02 carriers were found in 44.73% (17/38) and 40.00% (6/15) cases of CBZ and LTG-treated patients. It bears noting that the HLA-B*15:02 allele frequency was higher (66.67%) in OXC-induced cADRs, although the patient samples were too small to draw any definitive conclusions. However, there is evidence in the literature of the involvement of the HLA-B*15:02 allele in the OXC-induced SJS/TEN in populations in the Taiwan and Thailand [20]. Moreover, our data also support the Clinical Pharmacogenetics Implementation Consortium (CPIC) guideline for *HLA* genotype and use of CBZ and OXC [21].

In the present study, we support the hypothesis that multiple *HLA* alleles might be involved in AEDs-induced cADRs. Interestingly, HLA-B*51:01 was also found significantly associated with AEDs-induced cADRs with OR 2.292, but when we separated the data in each drug category, we found that HLA-B*51:01 was significantly associated with PHT-induced STS/TEN and CBZ-induced DRESS compared to tolerant controls with OR of 6.19 and 7.94, respectively. SJS/TEN caused by PHT and PB was significantly associated with the HLA-B*51:01 allele in the Japanese population [22]. Moreover, the carrier rate of HLA-B*13:01 in the PB-induced SCARs was 100% (*n* = 4). This study first demonstrated the association between HLA-B*13:01 and PB-induced SCARs in Thai adults [23]. Further, we corroborated the findings of Li et al. where patients carrying the HLA-B*58:01 allele were associated with CBZ-induced MPE [24]. We identified significant associations between CBZ-induced MPE and the HLA-B*58:01 allele, compared with CBZ-tolerant: OR 5.15, 95%CI: 1.64–16.14; *p* = 0.011 and compared with healthy subjects: OR 3.29, 95%CI: 1.10–9.82; *p* = 0.041(Data not shown).

In addition, the HLA-B*15:02 allele has been reported to be specifically associated with CBZ-induced SJS/TEN in Asian populations, and no associations have been reported for drug-induced MPE and DRESS [2, 8, 16, 25, 26]. In this study, we found significant associations between HLA-B*15:02 and AEDs-induced MPE and SJS/TEN, however, this significance was not observed in DRESS cases [27]. Interestingly, previous studies reported no associations between the HLA- B*15:02 allele and the AEDs-induced MPE [14, 28, 29]. We believe that further studies need to be conducted to explore the molecular mechanism explaining the linkage between the HLA-B*15:02 allele and MPE. In the SJS/TEN group, we identified the HLA-B*15:21 allele associated with AEDs-induced SJS/TEN (compared with healthy population: OR 10.636, 95%CI: 1.462–77.359, *p* = 0.042). The HLA-B*15:21 allele belongs to the group of HLA-B75 serotype which consists of HLA-B*15:02, HLA-B*15:08, HLA-B*15:11, and HLA-B*15:21. As evident with the previous findings, the HLA-B*15:02 allele was the genetic risk factor for an AEDs-induced SJS/TEN in our study. Interestingly, the HLA-B*15:11 allele, another member of the HLA-B75 serotype, was found in SJS/TEN cases due to CBZ [25, 30–34]. These findings suggest that HLA-B75 serotype might have a role in AEDs-induced SJS/TEN, suggesting the need for screening of other alleles of the HLA-B75 serotype in case of HLA-B*15:02 negative screening. In DRESS group, although we found positive correlations between AEDs-induced DRESS and HLA-B*08:01, HLA-B*13:01, and HLA-B*56:02 alleles. If Bonferroni correction was performed for multiple comparisons, the significance of these alleles would be lost.

In the PHT group, we found similar frequency of the HLA-B*15:02 allele between the cases and the controls (19.40% *vs* 17.10%). This result suggests that the patient carrying HLA-B*15:02 allele is not at risk of PHT-induced cADRs, this finding contradicts that of Dean L., who recommended avoiding PHT as an alternative for CBZ in patients positive for HLA-B*15:02 [19]. In addition to PHT, we further genotyped the CYP2C9*3 variants in 92 cases of PHT-induced cADRs and found that only 9 patients showed *CYP2C9* variation (4 SJS/TEN, 4 DRESS, and 1 MPE). No signification between CYP2C9*3 and PHT-induced cADRs. There was a trend that the carrier rates of CYP2C9*3 were higher in the PHT-induced SJS/TEN group than in the tolerant control group in this study. However, previous studies demonstrated that CYP2C9*3 and having Chinese ancestry were significant risk factors of PHT-induced SJS (adjusted OR = 5.40, *p* = 0.0097). [35–38].

HLA-A* 31:01 also showed to increase the risk of MPE and DRESS due to CBZ in European and Han-Chinese population [21]. Therefore, we analyzed the *HLA-A* in patients with CBZ-induced DRESS (*n* = 5) and CBZ-induced MPE (*n* = 16). Surprisingly, HLA-A*31:01 was not found in the patients with DRESS and MPE groups, suggesting that the HLA-A*31:01 allele could not be associated in the development of CBZ induced MPE and DRESS in Thai population. However, there was a trend that carriers rate of HLA-A*33:03 were higher in the MPE and DRESS groups than in the tolerant group. HLA-A*33:03 allele was detected in 60.00% of CBZ-induced DRESS (3/5) and 37.5% of CBZ-induced MPE (6/16). The HLA-A*31:01 is very low frequency (allele frequency=0.0085) but HLA-A*33:03 is the most common *HLA-A* alleles in Thai (allele frequency = 0.1117) [39]. In addition, a previous sequence analysis showed that HLA-A*31:01 and HLA-A*33:03 binding peptides have the same anchor residues at P2 and the C-terminus, the only difference being that HLA-A*33:03 binding peptides have two additional P2 anchor residues [40].

Finally, the meta-analysis was performed to clarify the relationship between HLA-B*15:02 and cADRs. There was a significant association between HLA-B*15:02 and AEDs-induced cADRs, and AEDs-induced SJS/TEN. while the association between HLA-B*15:02 and AEDs-induced DRESS did not reach statistical significance.

In conclusion, we found the association between HLA-B*15:02 and AEDs-induced cADRs. We also revealed an ambiguous association between the HLA-B*15:02 and the AEDs. Our results suggest that HLA-B*15:02 is not a risk allele for the cross-reactivity of AEDs-induced cADRs. We did not find any significant association between HLA-B*15:02 and PHT and PB-induced cADRs. If a patient has a positive HLA-B*15:02 genotype, a prescription for OXC needs to be carefully considered.
